# Disappearance of Cdc14 from the daughter spindle pole body requires Glc7-Bud14

**DOI:** 10.55730/1300-0152.2707

**Published:** 2024-09-17

**Authors:** İdil KIRDÖK, Ayşe KOSA ÇAYDAŞI

**Affiliations:** Department of Molecular Biology and Genetics, College of Sciences, Koç University, İstanbul, Turkiye

**Keywords:** Cdc14, protein phosphatase 1, Bud14, spindle pole body, mitotic exit network

## Abstract

**Background/aim:**

The conserved phosphatase Cdc14 facilitates mitotic exit in budding yeast by counteracting mitotic cyclin-dependent kinase activity. Cdc14 is kept in the nucleolus until anaphase onset, when it is released transiently into the nucleoplasm. In late anaphase, Cdc14 is fully released into the cytoplasm upon activation of the mitotic exit network (MEN) to trigger mitotic exit. Cdc14 also localizes to yeast spindle pole bodies (SPBs) in anaphase and dephosphorylates key targets residing on SPBs to allow SPB duplication and prime the MEN. Protein phosphatase 1 (Glc7) with regulatory subunit Bud14 is another phosphatase that plays a key role in the spatiotemporal control of mitotic exit. In this study, we investigated the regulation of Cdc14 localization by Bud14-Glc7.

**Materials and methods:**

We used fluorescence microscopy to analyze Cdc14 localization in *BUD14* wildtype and *BUD14* knockout cells (*bud14Δ*) as well as in cells expressing a mutant allele of *BUD14* (*bud14-F379A*) that cannot bind Glc7. We also utilized a yeast two-hybrid (Y2H) system to examine the interaction of Bud14 with Cdc14.

**Results:**

We found that Cdc14 remains at the SPBs longer in *bud14Δ* and *bud14-F379A* compared to wildtype cells. This effect is limited to the SPB that has migrated to the daughter cell (dSPB). Cdc14 localizes to both SPBs shortly after anaphase onset. In mid-to-late anaphase, levels of Cdc14 increase at the dSPB in both wildtype and *bud14Δ* cells. With mitotic exit, Cdc14 disappears from the dSPB in wildtype cells but not in *bud14Δ* cells. Accordingly, 50% of *bud14Δ* cells in G1 have Cdc14 at their SPBs. We also found that Cdc14 localization at the dSPB was largely, but not entirely, dependent on Bfa1 in *bud14Δ* cells. Furthermore, Bud14 interacted with Cdc14 in the Y2H system.

**Conclusion:**

Our results suggest that Glc7-Bud14 is part of a mechanism that promotes Cdc14 disappearance from the dSPB.

## 1. Introduction

Cdc14 is a conserved single-subunit proline-directed dual-specificity protein phosphatase, first discovered in the budding yeast *Saccharomyces cerevisiae* (Visintin et al., 1998; Gray et al., 2003; Partscht and Schiebel, 2023). Cdc14 facilitates mitotic exit in budding yeast by downregulating mitotic cyclin-dependent kinase (CDK) activity and by dephosphorylating CDK substrates (Visintin et al., 1998). Until anaphase onset, Cdc14 is kept sequestered in the nucleolus as a result of binding to Cfi1/Net1 (Visintin et al., 1999). In early anaphase, Cdc14 is transiently and partially released from the nucleolus through the Cdc fourteen early anaphase release (FEAR) pathway (Stegmeier et al., 2002). In late anaphase, upon activation of the mitotic exit network (MEN), Cdc14 is fully released from the nucleolus into the cytoplasm, which is essential for mitotic exit (Jaspersen et al., 1998; Shou et al., 1999). While partial release of Cdc14 is not essential for mitotic exit, it governs anaphase-related tasks such as the positioning of the nucleus, anaphase spindle stabilization, and ribosomal RNA segregation as well as priming of the MEN (Manzano-Lopez and Monje-Casas, 2020; Partscht and Schiebel, 2023).

In addition to its key roles in mitotic exit, rDNA segregation, and anaphase spindle stabilization, Cdc14 is also critical for many other cellular processes including the regulation of the duplication of spindle pole bodies (SPBs), the yeast equivalent of centrosomes, by dephosphorylating the SPB half-bridge protein Sfi1 to allow new SPB duplication in anaphase (Elserafy et al., 2014). Cdc14 localizes to the SPBs after its release by the FEAR network in both mitosis and meiosis (Pereira et al., 2002; Fox et al., 2017). SPB association of Cdc14 is likely to be important for its roles at the SPB. Targets of Cdc14 that reside on SPBs include Bfa1, Cdc15, and Sfi1, associated with the roles of Cdc14 in priming the MEN and allowing SPB duplication (Jaspersen and Morgan, 2000; Pereira et al., 2002; Elserafy et al., 2014).

Other phosphatases with key mitotic functions also communicate with Cdc14. For example, protein phosphatase 2A (PP2A) with regulatory subunit Cdc55 inhibits partial release of Cdc14 by directly dephosphorylating Cfi1/Net1 (Queralt et al., 2006). Furthermore, PP2A with regulatory subunit Rts1 and protein phosphatase 1 (Glc7) with regulatory subunit Bud14 are essential components of the spindle position checkpoint (SPOC), which inhibits the MEN in response to spindle alignment defects, thus indirectly inhibiting full release of Cdc14 in cells with mispositioned spindles (Chan and Amon, 2009; Kocakaplan et al., 2021). Interestingly, Glc7-Bud14 is a regulator of the mitotic exit inhibitor Bfa1, which is a protein crucial for Cdc14 localization at SPBs (Kocakaplan et al., 2021). However, whether or not Glc7-Bud14 impacts the SPB localization of Cdc14 remains unknown.

In this study, we began by asking whether Glc7-Bud14 affects Cdc14 release in cells with correctly positioned spindles. We show that partial and full release of Cdc14 remains unchanged in cells lacking *BUD14* (*bud14Δ*). However, unexpectedly, we discovered that Cdc14 localization at the SPBs is prolonged in *bud14Δ* cells as well as in *bud14-F379A* mutants that cannot bind to Glc7. Through fluorescence intensity measurements, we found that this effect is limited to the SPBs that migrated to daughter cells (dSPBs). Cdc14 localizes to both SPBs shortly after anaphase onset, whereas in mid-to-late anaphase it increases at the dSPB in both wildtype and *bud14Δ* cells. With mitotic exit, Cdc14 disappears from the SPBs in wildtype cells but not in *bud14Δ* cells. Accordingly, 50% of the *bud14Δ* cells in G1 have Cdc14 at their SPBs. We further show that Cdc14 localization at the dSPB is largely, but not entirely, dependent on Bfa1 in *bud14Δ* cells. Our results thus suggest a novel role for Glc7-Bud14 in Cdc14 disappearance from the dSPB. In support of this, we show that Bud14 interacts with Cdc14 in a yeast two-hybrid (Y2H) system.

## 2. Materials and methods

### 2.1. Yeast strains and growth conditions

The utilized yeast strains are listed in Table 1. All selected yeast strains are isogenic to S288C. Growth media were prepared as described by Sherman (1991). Cells were grown at 30 °C unless otherwise stated. For routine cell growth, YPAD media or YPD agar plates were used. Synthetic complete (SC) media that lacked the respective auxotrophic nutrients (SC-x) were used for the growth of cells containing Y2H plasmids. For microscopy-based experiments, SC media were used. All media and agar plates were autoclaved except for those used in microscopy-based experiments, for which the media were filter-sterilized.

For G1 synchronization of cells, log-phase cultures were incubated for 1.5 doubling time in the presence of 10 μg/mL α-factor (#T6901, Sigma, St. Louis, MO, USA). Arrest was confirmed by microscopy.

For deletion and C-terminal tagging of genes on the chromosome, cassette PCR-based methods were used (Janke et al., 2004). Transformants were selected on SC-x or antibiotic-containing YPD agar plates depending on the selection marker. For visualization of tubulin, mCherry-*TUB1-*containing integration plasmids were integrated into the appropriate chromosomal loci (*URA3* or *LEU2*) and transformants were selected on appropriate SC-x agar plates. Plasmids used in this study are listed in Table 2. Gene deletions were confirmed by PCR, whereas C-terminal tagging was confirmed by immunoblotting and/or microscopy.

### 2.2. Fluorescence microscopy

Fluorescence microscopy was performed using an Axio Observer-7 motorized inverted epifluorescence microscope with a Colibri 7 LED light source, Axiocam 702 Monochrome camera, and 100X Plan-Apochromat objective (Carl Zeiss, Oberkochen, Germany). The filter sets were #95 and #44. Cells were fixed using 8% paraformaldehyde (30525-89-4, Merck, Darmstadt, Germany) and analyzed by microscopy within the next 24 h. Fixed cell suspensions were sandwiched between slides and coverslips for imaging. Images of 10 z-stacks with intervals of 0.36 μm were acquired for each position.

### 2.3. Image analysis

All image analyses were performed using ImageJ software (National Institutes of Health, Bethesda, MD, USA) (Schneider et al., 2012). The z-stacks were projected using the max-projection method. For cell percentage calculations, Cdc14 partial release, Cdc14 full release, and SPB localization were assessed by eye and spindle length was measured using the line selection tool in ImageJ. For SPB-bound Cdc14-GFP fluorescence intensity measurements, an area of 0.343 μm^2^ (25 pixels) around the tips of the spindle was selected using the rectangular selection tool and mean gray values were measured at the GFP channel using the measurement tool of ImageJ. For each cell, a mean gray value of an area within the cytoplasm devoid of SPBs was measured as the background signal. The background was subtracted from the SPB signal to obtain the corrected mean signal intensities.

Graphs and statistical results were obtained using GraphPad Prism 8.0.1 (GraphPad Inc., La Jolla, CA, USA). Images were adjusted for brightness and contrast, compiled together, and labeled using Photoshop and Illustrator 2024 (Adobe, San Jose, CA, USA).

### 2.4. Yeast two-hybrid assay

The pMM5 and pMM6 plasmid pairs containing the gene of interest were transformed into SGY37-VIII,3. Stationary cultures of SGY37-VIII,3 bearing the pMM5/pMM6 plasmid pairs were diluted to 1.0 OD_600_ and 10 μL from each culture was dropped onto agar plates. After 1–2 days of incubation, plates were overlaid with soft agar (0.4%) solution as previously described (Kocakaplan et al., 2021).

### 2.5. Protein methods

Total protein extraction, SDS-PAGE, and immunoblotting were performed as previously described (Kocakaplan et al., 2021). The primary antibodies were mouse anti-HA (a gift from Gislene Pereira) and rabbit anti-tubulin (EPR13799, Abcam, Cambridge, UK). The horseradish peroxidase-conjugated secondary antibodies were goat antirabbit (#R-05072-500, Advansta, San Jose, CA, USA) or goat antimouse (#R-05071-500, Advansta). Chemiluminescence was detected using an enhanced chemiluminescence solution (ECL, 0.1 M Tris (pH 8.5), 1.25 mM luminol, 0.2 mM p-coumaric acid, 0.01% H_2_O_2_) with the ChemiDoc MP imager (Bio-Rad, Hercules, CA, USA).

## 3. Results

### 3.1. Bud14 does not affect the full release of Cdc14 in unperturbed cell cycles

**Figure 1:**
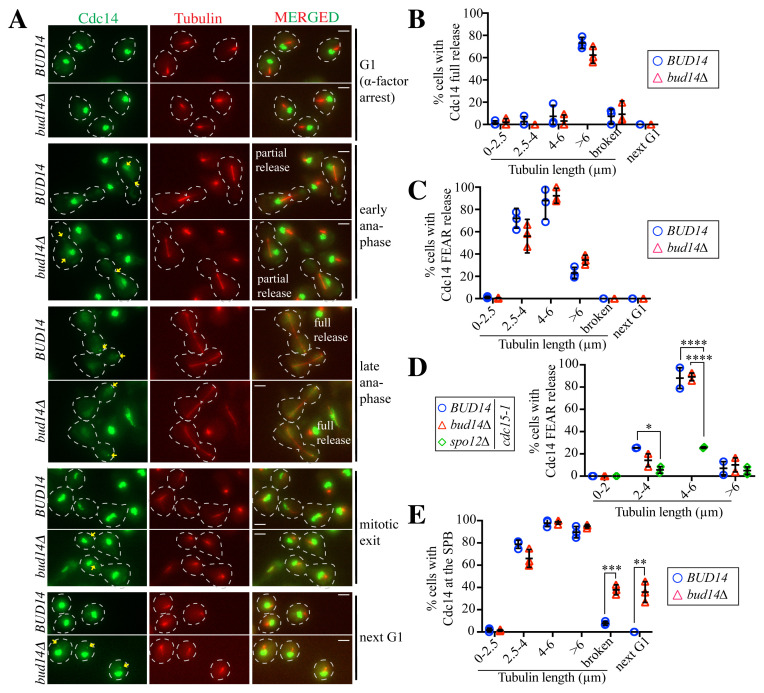
Bud14 does not affect Cdc14 full and partial release in an unperturbed cell cycle **A)** Representative images for Cdc14 release and localization at SPBs. Scale bars: 3 μm. Cells in the indicated categories are outlined. Arrows mark Cdc14 at SPBs. **B–D)** Percentages of cells with Cdc14 full release (B) and partial release (C, D). **E)** Percentages of cells with Cdc14 at SPBs. Replicates are shown as individual data points. The graphs in B, C, and E show the results of three independent experiments, whereas the graph in D shows two independent experiments. For each data point at least 100 cells were counted per experiment. Mean values and standard deviations of independent experiments are indicated. The unpaired multiple t-test was used to determine significance in the graphs in B, C, and E. Two-way ANOVA was conducted to determine significance in D. *: p < 0.05, **p < 0.01, ***: p < 0.001, ****: p < 0.0001.

In response to spindle positioning defects, Bud14 acts as an inhibitor of mitotic exit (Kocakaplan et al., 2021). We aimed to determine whether Bud14 also impinges on mitotic exit in cells with correctly positioned spindles. For this, we analyzed Cdc14 full release as an indicator of mitotic exit. To quantify the timing of Cdc14 full release with respect to anaphase onset, we synchronized mCherry-Tub1 Cdc14-GFP-bearing wildtype and *bud14Δ* cells in the G1 phase with α-factor treatment. Subsequently, cells were released from the G1 block to allow synchronous cell cycle progression and samples were collected for microscopy every 10 min for one complete cell cycle. Using mCherry-Tub1 as a spindle marker and measuring spindle length in cells, we grouped cells as being in G1 to metaphase (spindles of 0–2.5 μm), early anaphase (spindles of 2.5–4 μm), middle anaphase (spindles of 4–6 μm), and late anaphase (spindles of >6 μm). Furthermore, cells that underwent spindle breakdown were categorized as cells that had exited mitosis but had not yet finalized cytokinesis/abscission (broken spindles), whereas cells that finished cytokinesis/abscission were grouped within the separate category of “next G1.” As expected, during α-factor-induced G1 arrest, Cdc14 was nucleolar in both wildtype (*BUD14*) and *bud14Δ* cells (Figure 1a). Cdc14 full release occurred in late anaphase (spindle length of >6 μm) in both *BUD14* and *bud14Δ* cells (Figures 1a and 1b) with no significant difference (Figure 1b). We thus concluded that Bud14 does not affect the timing of Cdc14 full release in cells with correctly positioned mitotic spindles.

### 3.2. Bud14 does not affect partial release of Cdc14

We next analyzed partial release of Cdc14 in the same experimental set-up used for Cdc14 full release (Figures 1a and 1c). The dynamics of partial release were similar in the wildtype (*BUD14*) and *bud14Δ* cells in these experiments (Figures 1a and 1c). Because the FEAR pathway is followed by mitotic exit in cells with an intact MEN, analysis of the FEAR dynamics of MEN mutants allows a more precise assessment of the Cdc14 released by the FEAR network (Pereira et al., 2002). Thus, we also analyzed Cdc14 partial release in *cdc15-1* cells, in which MEN is inhibited at the restrictive temperature (37 °C) (Jaspersen et al., 1998) (Figure 1d). *cdc15-1* and *cdc15-1 bud14Δ* cells were synchronized in G1 using α-factor at 23 °C. The cells were then released from G1 arrest at 37 °C. *cdc15-1 spo12Δ* cells were also analyzed as control cells defective in FEAR (Stegmeier et al., 2002). Cdc14 release by the FEAR network started in early anaphase, peaked during middle anaphase, and decreased in late anaphase in *cdc15-1* cells (Figure 1d). *cdc15-1 bud14Δ* cells behaved similarly to *cdc15-1* cells (Figure 1d), consistent with the previous results obtained for MEN-uninhibited cells (Figure 1c). The Cdc14 partial release observed in both *cdc15-1* and *cdc15-1 bud14Δ* was significantly different compared to the findings for FEAR-deficient *cdc15-1 spo12Δ* cells (Figure 1d). Taken together, these experiments suggest that Bud14 does not impact partial or full release of Cdc14 in cells with correctly positioned spindles.

### 3.3. Spindle pole body localization of Cdc14 is prolonged in *bud14Δ* cells

Although partial and full release of Cdc14 were not significantly affected by *BUD14* deletion, we noticed a drastic change in the localization of Cdc14 at SPBs (Figures 1a and 1e). In wildtype cells synchronously released from α-factor-induced G1 arrest, Cdc14 localized to the SPBs after anaphase onset (spindle length: 2.5–4 μm) and remained at the SPBs until late anaphase (spindles of >6 μm) (Figures 1a and 1e). In *bud14Δ* cells, Cdc14 was detected at the SPBs after anaphase onset, as was the case for the wildtype cells. However, surprisingly, Cdc14 did not decrease on SPBs at late anaphase; rather, it remained there after mitotic exit (broken spindles) and during the next G1 phrase (Figures 1a and 1e). These data indicate that Bud14 may be part of a mechanism that promotes Cdc14 dissociation from SPBs.

**Figure 2:**
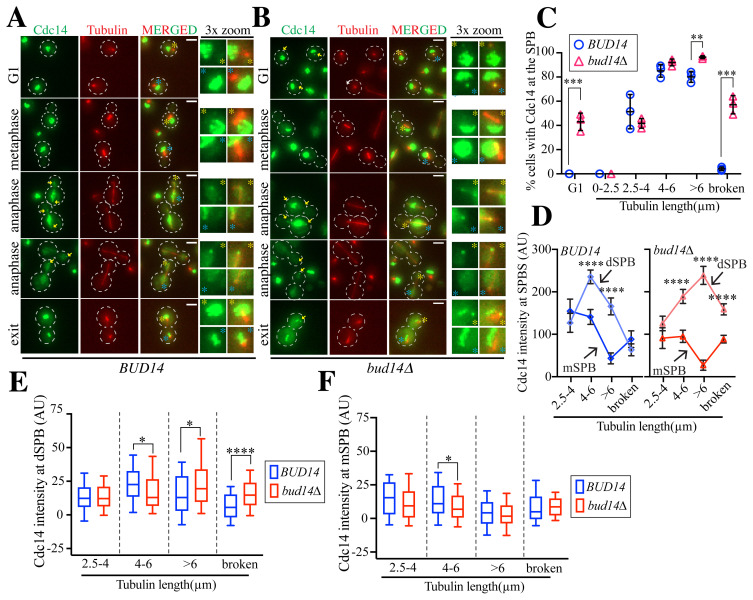
Cdc14 localization to the SPBs is prolonged in *bud14Δ* until entry into the subsequent cell cycle **A, B)** Representative images for Cdc14 SPB localization in wildtype (A) and *bud14Δ* (B) cells. Cells were obtained from logarithmically growing cultures. Arrows mark Cdc14 at SPBs. Threefold enlargements show the SPBs marked with yellow and blue asterisks. Tubulin asters or spindle tips serve as references for SPB. Scale bars: 3 μm. **C)** Percentage of cells with Cdc14 SPB localization. Results from three independent experiments are shown as individual data points. For each data point, at least 100 cells were counted per experiment. Mean values and standard deviations of independent experiments are shown. **D, F)** Fluorescence intensities of Cdc14 at mSPBs and dSPBs in wildtype (*BUD14*) and *bud14Δ* cells. A minimum of 100 SPBs were quantified for each spindle length interval except for 2.5–4 μm, for which 30–40 SPBs were quantified. The mean values and standard errors of the means are shown in D. Box-and-whisker plots are provided in E and F. The boxes indicate the 10th and 90th percentiles while whiskers show the minimum and maximum values. The lines inside the boxes signify the mean values. The unpaired t-test was used to determine significance. *: p < 0.05, **p < 0.01, ***: p < 0.001, ****: p < 0.0001.

Puzzlingly, although Cdc14 was present at the SPBs of *bud14Δ* cells in the next G1 phase, it did not localize to the SPBs during α-factor-induced G1 arrest (Figures 1a and 1e). Because the SPB size and structure change during the α-factor arrest (Pereira et al., 1999; Yoder et al., 2003), we reasoned that this discrepancy might have resulted from α-factor-induced changes in the SPBs. Therefore, to avoid any influence of α-factor on our results, we analyzed the SPB localization of Cdc14 in logarithmically growing asynchronous cells (Figures 2a–2c). Cells were grouped according to spindle length as described for the experiment that utilized α-factor synchronization. In line with the results from the α-factor release experiment, Cdc14 localized to the SPBs in wildtype cells after anaphase onset (spindles of 2.5–4 μm) and persisted at the SPBs until late anaphase (spindles of >6 μm) (Figures 2a and 2c). While none of the unbudded wildtype cells had Cdc14 at their SPBs (Figures 2a and 2c), Cdc14 localized at the SPBs in ~50% of no budded (G1) cells (Figures 2b and 2c). Cdc14 disappeared from the SPBs soon after the *bud14Δ* cells started budding (spindles of 0–2.5 μm) (Figures 2b and 2c). After anaphase onset, Cdc14 relocalized to the SPBs of *bud14Δ* cells, similar to wildtype cells (Figures 2b and 2c). After mitotic exit (broken spindles), Cdc14 localization at the SPBs persisted in *bud14Δ* cells, although it disappeared from the SPBs of wildtype cells. In concordance with the experiment that used α-factor synchronization, this experiment supported the conclusion that Cdc14 does not disappear from the SPBs of *bud14Δ* cells after mitotic exit; rather, it remains at the SPBs until the next G1/S phase.

### 3.4. Cdc14 fails to dissociate from the dSPB in *bud14Δ* cells

To understand Cdc14 SPB localization in more detail, we measured relative levels of Cdc14-GFP at the SPBs in wildtype (*BUD14*) and *bud14Δ* cells (Figures 2d–2f). In this analysis, we distinguished Cdc14 localization at the dSPBs, which are directed towards the bud, and the mother SPBs (mSPBs), which remain in the mother cell compartment (Figures 2d and 2f). In both wildtype and *bud14Δ* cells during early anaphase (spindles of 2.5–4 μm spindle), the Cdc14-GFP levels at the dSPBs and mSPBs were similar (Figure 2d). Of note, these cell types had similar levels of Cdc14 at their SPBs in early anaphase (Figures 2e and 2f). During middle anaphase (spindles of 4–6 μm) and late anaphase (spindles of >6 μm), more Cdc14 was present at the dSPBs than the mSPBs in both types of cells (Figure 2d). In middle anaphase (spindles of 4–6 μm) the *bud14Δ* cells had slightly lower levels of Cdc14 at the SPBs than the wildtype cells, but this situation was reversed in late anaphase at the dSPBs (Figures 2e and 2f). Notably, Cdc14 disappeared from the mSPBs during late anaphase (spindles of >6 μm) in both cell types (Figures 2d and 2f). Consistent with our previous results, the Cdc14 signal remained at the dSPBs of *bud14Δ* cells after mitotic exit (broken spindles), whereas it disappeared in wildtype cells (Figures 2d and e). These findings suggest that only the daughter cells inherit the SPB-localized Cdc14 in *bud14Δ* cells after the completion of cell division, which is in line with our data revealing that approximately half of the unbudded *bud14Δ* cells had Cdc14-GFP at their SPBs (Figure 2c). Taken together, these results show that the Bud14-dependent change in Cdc14 SPB localization is largely restricted to the dSPB. Given that Bud14 localizes to the daughter cell cortex (Cullen and Sprague, 2002; Knaus et al., 2005), Bud14 is likely to act on Cdc14 in the daughter cells.

### 3.5. Bud14 interaction with protein phosphatase 1 is required for Cdc14 removal from SPBs after mitotic exit

**Figure 3:**
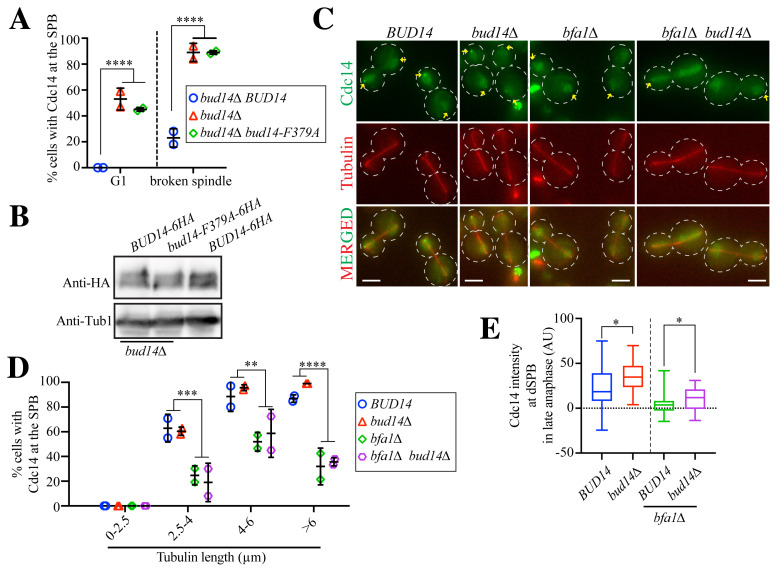
Cdc14 localization to the SPBs are influenced by Bud14-Glc7 interaction and Bfa1 **A)** Percentage of cells with Cdc14 localized at the SPBs. Experiments were repeated twice and the mean values of each independent experiment are shown in the graph as individual data points. At least 100 cells were counted for each data point. The means and standard deviations of are indicated. **B)** Immunoblot showing the protein levels of Bud14–6HA, Bud14-F379A-6HA, and endogenous Bud14–6HA. Tub1 served as the loading control. **C)** Representative images of the experiments shown in D and E. Scale bar: 3 μm. Arrows indicate SPB-localized Cdc14. **D)** Percentage of cells with Cdc14 at the SPBs. Experiments were repeated twice and the mean values of each independent experiment are shown in the graph as individual data points. At least 100 cells were counted for each data point. The means and standard deviations are indicated. **E)** Fluorescence intensity of Cdc14-GFP at dSPBs in late anaphase (spindle length of >6 μm). For each cell type, 30–32 SPBs were quantified. Statistical methods were two-way ANOVA for A and D and the unpaired t-test for E. *: p < 0.05, **p < 0.01, ***: p < 0.001, ****: p < 0.0001.

Bud14 is one of the many regulatory subunits of Glc7, the protein phosphatase 1 present in budding yeast (Cullen and Sprague, 2002). We aimed to determine whether the influence of Bud14 on Cdc14 requires Bud14-Glc7 interaction. For this, we analyzed the SPB localization of Cdc14 in *bud14Δ* cells, which were supplemented with either the wildtype *BUD14* or a mutant allele of *BUD14* that cannot bind to Glc7 (*bud14-F379A*) (Knaus et al., 2005). The introduction of *BUD14* but not *bud14-F379A* rescued the SPB localization phenotype of the *bud14Δ* cells (Figures 3a and 3b). Thus, we concluded that Bud14 works together with Glc7 in the regulation of Cdc14 SPB localization.

### 3.6. Cdc14 SPB localization is largely dependent on Bfa1 in wildtype and *bud14Δ* cells

Upon its release from the nucleolus during early anaphase, Cdc14 is localized to SPBs via its interaction with the Bfa1-Bub2 complex (Pereira et al., 2002). Accordingly, Cdc14 SPB localization is Bfa1-dependent in wildtype cells (Pereira et al., 2002). We aimed to determine whether the Cdc14 SPB localization in *bud14Δ* cells is also Bfa1-dependent. To address this question, we quantified the percentage of cells with Cdc14 SPB localization in the presence or absence of *BFA1* and/or *BUD14* (Figures 3b and 3c). Upon the deletion of *BFA1* (*bfa1Δ*), Cdc14 SPB localization was greatly reduced in both wildtype (*BUD14*) and *bud14Δ* cells, but not completely diminished (Figures 3c and 3d). To compare the relative levels of SPB-bound Cdc14, we measured Cdc14-GFP signal intensities at the dSPBs of cells in late anaphase. We noticed that in both the absence and presence of *BFA1*, *bud14Δ* cells had slightly increased levels of Cdc14 at the dSPB during late anaphase (Figures 3c and 3e). These data suggest that although Cdc14 SPB localization is largely dependent on Bfa1, there is probably a small pool of Cdc14 that is able to localize to the dSPB in the absence of Bfa1, and this pool may be affected by Bud14.

### 3.7. Cdc14 interacts with Bud14 in the yeast two-hybrid system

**Figure 4:**
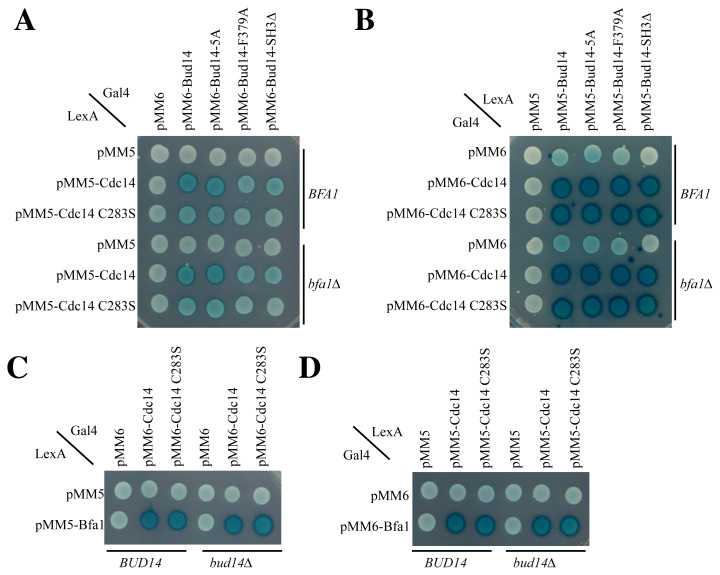
Cdc14 interacts with Bud14 in Y2H **A, B)** Cdc14–Bud14 Y2H assays. **C, D**) Cdc14–Bfa1 Y2H assays. One representative image from at least two independent experiments is shown.

Using the Y2H approach, we aimed to determine whether Bud14 is capable of interacting with Cdc14. Cdc14 and Bud14 were expressed in fusion with the LexA DNA-binding domain or Gal4 activation domain. The formation of a blue color indicates the interaction of Gal4 activation and the LexA DNA-binding domain, which happens when the proteins fused with them come into close proximity of each other. The Bud14–Cdc14 pair produced a blue color in both directions, indicating their proximity (Figures 4a and 4b). Furthermore, the Bud14-5A mutants that cannot bind formin, the Bud14-F379A that cannot bind Glc7, and the Bud14-SH3Δ mutant that cannot bind to the cortex (Knaus et al., 2005; Eskin et al., 2016) also interacted with Cdc14 in the Y2H system (Figures 4a and 4b). The entrapment mutant of Cdc14 (Cdc14-C283S) (Bloom et al., 2011) also interacted with Bud14, similar to the wildtype Cdc14 (Figures 4a and 4b).

As both Cdc14 and Bud14 are known to bind to Bfa1 (Pereira et al., 2002; Kocakaplan et al., 2021), we asked whether the Cdc14–Bud14 interaction is Bfa1-dependent. We accordingly analyzed the interactions against a *bfa1Δ* background. The interactions remained the same in the absence of *BFA1* in our Y2H system (Figures 4a and 4b), indicating that Cdc14–Bud14 interactions are not Bfa1-dependent. We next asked whether Bud14 impacts Cdc14–Bfa1 interaction, which could in turn affect Cdc14 SPB localization. For this, we analyzed Cdc14–Bfa1 Y2H interactions in the presence and absence of Bud14 (Figures 4c and 4d). The Cdc14–Bfa1 interaction remained the same in the *bud14Δ* cells (Figures 4c and 4d). These experiments suggest that Bud14 and Cdc14 interact with Bfa1 independently of each other and, likewise, Cdc14–Bud14 interactions are not dependent on Bfa1.

## 4. Discussion

Cdc14 is a conserved phosphatase with key roles during the cell cycle. In budding yeast, Cdc14 localizes to several cellular components in a cell cycle-dependent manner, including the nucleolus, nucleus, SPBs, cytoplasm, and bud neck, dephosphorylating their substrates. Although the regulation of Cdc14 nucleolus localization is well studied, little is known about how Cdc14 localization to other compartments is controlled. In the present study, we report that Glc7-Bud14 is part of a mechanism that promotes the disappearance of Cdc14 from the dSPB.

Our results indicate that Cdc14 remains longer at the SPBs in *bud14Δ* cells and the *bud14-F379A* mutant that cannot bind to Glc7. Furthermore, we found that this effect was limited to the dSPB. Although Cdc14 disappears from the dSPB of wildtype cells during mitotic exit, it remains at the dSPB of *bud14Δ* cells until the next G1/S phase. Of importance, the *bud14Δ* cells exhibited wildtype-like kinetics for partial and full Cdc14 release from the nucleolus. Thus, the prolonged dSPB localization of Cdc14 in these cells is unlikely to stem from any altered timing of the FEAR pathway and the MEN.

In light of our finding that Bud14 interacted with Cdc14 in the Y2H system, it is tempting to speculate that Bud14-Glc7 may directly affect Cdc14, promoting Cdc14 dissociation from the dSPB. However, it is worth noting that we failed to detect Cdc14 in complex with Bud14 using immunoprecipitation techniques, which may indicate the transient nature of this interaction. Alternatively, Bud14-Glc7 may directly affect SPB structural proteins or other SPB-associated proteins that serve as Cdc14 docking sites on SPBs. In support of this, Bud14 was previously implicated in restricting the amount of SPB structural proteins incorporated into the SPBs, hence playing a role in the maintenance of SPB size (Girgin and Çaydaşı, 2024). Furthermore, Bud14 interacts with Bfa1 (Kocakaplan et al., 2021) and other SPB associated proteins (Hruby et al., 2011; Zhou et al., 2021). Importantly, the SPB localization of Cdc14 is highly dependent on Bfa1, which predominantly localizes to the dSPB (Pereira et al., 2002). Our data also support the conclusion that the majority of Cdc14 localization to the SPBs occurs through Bfa1. However, our results further suggest the presence of a Bfa1-independent pool of Cdc14 at the SPBs, which becomes more evident in the absence of Bud14. Therefore, we suggest that Bud14 in the daughter cells may cause the dissociation of the Bfa1-independent pool of Cdc14 from the dSPB. In support of this, Bud14 localizes to the daughter cell cortex and is therefore enriched in the daughter cells (Cullen and Sprague, 2002; Knaus et al., 2005), and the Cdc14–Bud14 Y2H interaction is not dependent on Bfa1. Further studies will shed light on the molecular mechanisms of Glc7-Bud14 in promoting the dissociation of Cdc14 from dSPBs.

The targets of Cdc14 at SPBs fall into two categories. First, the proteins of SPOC and MEN, including Bfa1, Cdc15, Dbf2, and Mob1, are dephosphorylated by Cdc14 to promote timely mitotic exit (Jaspersen and Morgan, 2000; Xu et al., 2000; Menssen et al., 2001; Mohl et al., 2009; Konig et al., 2010). Second, the SPB half-bridge protein Sfi1 is dephosphorylated by Cdc14 in late anaphase, which allows for SPB duplication in the next cycle by the removal of the Cdk1 phosphorylation of Sfi1, thus promoting conversion of the half-bridge to a bridge (Avena et al., 2014; Elserafy et al., 2014). Accordingly, in the absence of Cdc14 function, SPB duplication is delayed. Because the timing of MEN activation is not altered in the absence of Bud14 and the Cdc14 dSPB localization normally disappears after mitotic exit, the role of Bud14-Glc7 in the regulation of Cdc14 SPB localization is unlikely to be significant for mitotic exit regulation. However, we anticipate that the removal of Cdc14 from SPBs may be crucial for allowing the Cdk1 phosphorylation of Sfi1 and therefore for timely SPB duplication. Supporting this, *bud14Δ* cells have a delay in budding and SPB separation (Girgin and Çaydaşı, 2024). Nevertheless, more research is required to understand whether the delay in the SPB separation of *bud14Δ* cells is linked to delayed Cdc14 removal from the SPBs.

## Figures and Tables

**Table 1:** Yeast strains used in this study.

Strain Name	Description	Reference
ESM356	*MATa ura3-52 leu2Δ1 his3Δ200 trp1Δ63*	(Pereira and Schiebel, 2001)
YPH499	*MATa ura3-52 lys2-801amber ade2-101ochre trp1Δ63 his3Δ200 leu2Δ1*.	(Sikorski and Hieter, 1989)
SGY37- VIII,3	*MATa leu2 his3 trp1 ADE2 ura3-52::URA3-lexA-op-LacZ*	(Geissler et al., 1996)
IKY032	*ESM356 ura3-52::URA3-mCherry-TUB1 CDC14-GFP-KanMX6*	This study
IKY039	*ESM356 ura3-52::URA3-mCherry-TUB1 CDC14-GFP-KanMX6 bud14Δ::his3MX6*	This study
IKY233	*ESM356 ura3-52::URA3-mCherry-TUB1 CDC14-GFP-KanMX6 bfa1Δ::kITRP1*	This study
IKY234	*ESM356 ura3-52::URA3-mCherry-TUB1 CDC14-GFP-KanMX6 bud14Δ::His3MX6 bfa1Δ::kITRP1*	This study
IKY074	*YPH499 cdc15-1 leu2Δ1::LEU2-mCherry-TUB1 CDC14-GFP-KanMX6*	This study
IKY079	*YPH499 cdc15-1 leu2Δ1::LEU2-mCherry-TUB1 CDC14-GFP-KanMX6 bud14Δ::kITRP1*	This study
IKY080	*YPH499 cdc15-1 leu2Δ1::LEU2-mCherry-TUB1 CDC14-GFP-KanMX6 spo12Δ::natNT2*	This study
DKY122	*SGY37-VIII,3 bfa1Δ::natNT2*	This study
IKY251	*SGY37-VIII,3 bud14Δ::kITRP1*	This study
IKY298	*ESM356 ura3-52::URA3-mCherry-TUB1 CDC14-GFP-KanMX6 bud14Δ::his3MX6 leu2Δ1::LEU2*	
IKY299	*ESM356 ura3-52::URA3-mCherry-TUB1 CDC14-GFP-KanMX6 bud14Δ::his3MX6 leu2Δ1::LEU2-BUD14-6HA-klTRP1*	This study
IKY300	*ESM356 ura3-52::URA3-mCherry-TUB1 CDC14-GFP-KanMX6 bud14Δ::his3MX6 leu2Δ1::LEU2-bud14-F379A-6HA-kITRP1*	This study
IKY301-1	*ESM356 ura3-52::URA3-mCherry-TUB1 CDC14-GFP-KanMX6 bud14Δ::his3MX6 BUD14-6HA-klTRP1*	This study

**Table 2:** Plasmids used in this study.

Plasmid Name	Description	Reference
pMM6	p425-Gal1-Gal4-HA.	(Geissler et al., 1996)
pMM5	p423-Gal1-lexA-Myc.	(Geissler et al., 1996)
pHA69-1	pMM5-*BUD14*	(Kocakaplan et al., 2021)
pHA70-1	pMM6-*BUD14*	(Kocakaplan et al., 2021)
pCL4a-3	pMM6-*BFA1*	(Pereira et al., 2002)
pSM890a-1	pMM6-*CDC14*	(Pereira et al., 2002)
pSM993-1	pMM5-*CDC14*	(Pereira et al., 2002)
pCL3A-5	pMM5-*BFA1*	(Pereira et al., 2002)
pSP137-1	pMM6-Cdc14-C283S	(Pereira et al., 2002)
pSP132-1	pMM5-Cdc14-C283S	(Pereira et al., 2002)
IKY006	pMM5-*BUD14-5A*	(Kocakaplan et al., 2021)
IKY007	pMM5-*BUD14-F379A*	(Kocakaplan et al., 2021)
IKY008	pMM5-*BUD14-ΔSH3*	(Kocakaplan et al., 2021)
pSMG02-1	pRS405-BUD14	This study
pSMG06-1	pRS405-*BUD14-F379A*	This study
pRS405	LEU2-based yeast integration plasmid	(Sikorski and Hieter, 1989)
